# Authors’ Reply to: Comment on “Feasibility of a New Cuffless Device for Ambulatory Blood Pressure Measurement in Patients With Hypertension: Mixed Methods Study”

**DOI:** 10.2196/16205

**Published:** 2020-04-22

**Authors:** Sebastian J H Bredie, Jelske M de Jong, Tom H van de Belt, Harry van Goor

**Affiliations:** 1 Department of Internal Medicine Radboud University Medical Center Nijmegen Netherlands; 2 REshape Center for Innovation Radboud University Medical Center Nijmegen Netherlands; 3 Department of Surgery Radboud University Medical Center Nijmegen Netherlands

**Keywords:** cuffless blood pressure, pocket devices, ambulant blood pressure

We thank Noud van Helmond and colleagues [1] for the critical assessment of our study results.

In a number of studies, we have recently evaluated the possible value of a handy device (Viatom’s Checkme) for measuring multiple vital parameters, including cuffless blood pressure (BP) determination.

The Checkme device entered the medical domain after it was originally designed for the consumer market. This makes it very interesting and necessary to scientifically investigate its use in patients.

After a comparison with a common BP monitor [2] and an evaluation of the self-assessment results by admitted patients [3], we recently reported the results of its use in ambulatory BP measurement [4]. In all these studies, both quantitative and qualitative aspects of the use of the Checkme were scientifically assessed.

We are aware of the questions about validity and certification raised by van Helmond et al [1], and we are pleased that through this platform, we can discuss the issues that we have already covered extensively in our manuscripts. Regarding the validity of Checkme’s systolic BP results, we stated, as discussed extensively in our previous comment on their letter [5], that as long as there is no adequate validation protocol specifically for cuffless BP monitors, a formal validation study in accordance with leading protocols is impossible. Thus, in its current form, it is too early to implement a device such as Checkme in daily practice. We found that a real-life comparison currently gives the best insight into the potential value. In their study of both of a smartwatch and a portable health device (Checkme), van Helmond et al [6] concluded that the Bodimetrics device was more accurate, possibly due to calibration immediately prior to the study. However, the BP device still failed to meet the accuracy guidelines of the Association for the Advancement of Medical Instrumentation validation protocol, from which van Helmond et al derived their investigation. This protocol assumes that a device should actually be capable of measuring BP without an initial calibration reference measurement. This is peculiar, since it is precisely for the use of cuffless BP monitors that a validation measurement with a traditional BP monitor is required (for estimation of vascular compliance using pulse-oxymetry and electrocardiogram). Only then can an estimate of the BP with these two signals be made. The argument that accuracy improves after a validation measurement taken shortly before a cuffless measurement [6] is therefore not valid. Precisely, the choice of reference BP monitors and conditions under which measurement is to be made are not included in the current validation protocols and are the reason that regulatory authorities such as the US Food and Drug Administration could not release Checkme for BP measurement.

With regard to the interpretation of the accuracy of the absolute BP values in our home measurement study, we disagree with van Helmond et al [1]. The clinical practice of treating hypertension is increasingly based on home measurement of BP. Here, measurement variation due to patient and environmental factors is taken for granted by the practitioners, since titration of the treatment based on these home measurements ultimately has a better clinical outcome than treatment based on office measurements. Although the Checkme is user-friendly, disruptive factors such as those found in home measurements cannot be excluded. A comparison of home measurement with different devices illustrates this phenomenon and will never have a strong agreement. That is not our message either. It is all about obtaining many measurement results in order to titrate medical treatment. With only a few reports available, including the study by Schoot et al [2], it is too early to promote cuffless measurements on a large scale. However, devices that make use of this technique do appear to be useful at present in, for example, outpatient BP measurement.

Finally, we agree with van Helmond et al [1] that a major advantage of the Checkme is its user-friendliness. All participants were able to take the measurement (12 participants in total). As mentioned in the article, one participant was excluded because the calibration of Checkme did not succeed. The cause for this is unknown, but the measurement itself was performed correctly. Calibration failure was, however, observed in about 10% of the participants in our previous studies [2-4], and this may be a reason for not using Checkme. The cause for this issue is mostly unclear. If a patient could use the Checkme device, it became clear that he or she started measuring BP much more often and that this self-assessment was a positive point raised in the interviews.

We hope that our joint effort to scientifically examine these new devices leads to optimization of self-monitoring technology and eventually to improved patient care.

## Abbreviations

BP: blood pressure
